# The functional role of *CST1* and *CCL26* in asthma development

**DOI:** 10.1002/iid3.1162

**Published:** 2024-01-19

**Authors:** Angela Hoyer, Sandip Chakraborty, Ingrid Lilienthal, Jon R. Konradsen, Shintaro Katayama, Cilla Söderhäll

**Affiliations:** ^1^ Department of Women's and Children's Health Karolinska Institutet Solna Sweden; ^2^ Astrid Lindgren Children's Hospital Karolinska University Hospital Solna Sweden; ^3^ Childhood Cancer Research Unit, Department of Women's and Children's Health Karolinska Institutet Solna Sweden; ^4^ Department of Biosciences and Nutrition Karolinska Institutet Huddinge Sweden; ^5^ Stem Cells and Metabolism Research Program University of Helsinki Helsinki Finland; ^6^ Folkhälsan Research Center Helsinki Finland

**Keywords:** A549, allergic asthma, CCL26, CST1, OLINK, RNA sequencing

## Abstract

**Background:**

Asthma is the most common chronic disease in children with an increasing prevalence. Its development is caused by genetic and environmental factors and allergic sensitization is a known trigger. Dog allergens affect up to 30% of all children and dog dander‐sensitized children show increased expression of cystatin‐1 (*CST1*) and eotaxin‐3 (*CCL26*) in nasal epithelium. The aim of our study was to investigate the functional mechanism of *CST1* and *CCL26* in the alveolar basal epithelial cell line A549.

**Methods:**

A549 cells were transfected with individual overexpression vectors for *CST1* and *CCL26* and RNA sequencing was performed to examine the transcriptomics. edgeR was used to identify differentially expressed genes (= DEG, |log_2_FC | ≥ 2, FDR < 0.01). The protein expression levels of A549 cells overexpressing *CST1* and *CCL26* were analyzed using the Target 96 inflammation panel from OLINK (antibody‐mediated proximity extension–based assay; OLINK Proteomics). Differentially expressed proteins were considered with a |log_2_FC| ≥ 1, *p* < .05.

**Results:**

The overexpression of *CST1* resulted in a total of 27 DEG (1 upregulated and 26 downregulated) and the overexpression of *CCL26* in a total of 137 DEG (0 upregulated and 137 downregulated). The gene ontology enrichment analysis showed a significant downregulation of type I and III interferon signaling pathway genes as well as interferon‐stimulated genes. At the protein level, overexpression of *CST1* induced a significantly increased expression of CCL3, whereas *CCL26* overexpression led to increased expression of HGF, and a decrease of CXCL11, CCL20, CCL3 and CXCL10.

**Conclusion:**

Our results indicate that an overexpression of *CST1* and *CCL26* cause a downregulation of interferon related genes and inflammatory proteins. It might cause a higher disease susceptibility, mainly for allergic asthma, as *CCL26* is an agonist for CCR‐3‐carrying cells, such as eosinophils and Th2 lymphocytes, mostly active in allergic asthma.

## Graphical abstract

Our study examined the transcriptomics after overexpression of *CST1* and *CCL26* separately in the alveoloar basal epithelial cell line A549 and observed a significant downregulation of type I and III interferons as well as interferon‐stimulated genes. At protein level, the overexpression of *CST1* and *CCL26* induced a differentially expression of inflammatory proteins. Our results highlight the role of *CST1* and *CCL26* in asthma development due to their role in inflammation.

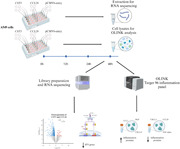

## 1 INTRODUCTION

Asthma is among the most common chronic diseases worldwide in children and adults. Onset is most often in childhood, and its prevalence has increased in recent years. The disease is characterized by chronic inflammation of the lower airways, variable airflow obstruction and airway hyperresponsiveness, leading to symptoms such as wheezing, shortness of breath and coughing. Wheezing episodes are mostly caused by rhinovirus infections that may result in a broken epithelial barrier and lead hereby more easily to inflammations.^1^, ^2^ Asthma can be divided into subgroups according to the type of inflammation driving the disease. Childhood asthma is divided into allergic asthma (Th2 inflammation), nonallergic and mixed, with allergic asthma being the most prevalent type of asthma in children.^3^ The disease leads to life‐long consequences for patients, including loss of time for work and education, impaired quality of life, and high costs for society.^4^ The most common asthma treatments nowadays are bronchodilators and inhaled corticosteroids, which act as airway inflammation suppressors due to the downregulation of pro‐inflammatory cytokines and inflammatory genes.^5^ The development of asthma stems from genetic and environmental factors. Though no exact genetic mechanisms are currently known, several genes have been suggested to be asthma susceptibility genes.^6^

Allergic sensitization is a known trigger for asthma development. One major cause are dog allergens which affect up to 30% of all children and adolescents.^7^ A Swedish birth cohort study including children between 4 and 16 years of age showed that allergic sensitization to dog is increasing from 4.8% to 22.6%.^8^ Another Swedish population‐based cohort study including children between 11 and 12 years of age showed a sensitization to dog allergens of 31.5%.^9^

In a recent study, we investigated nasal epithelium of 54 dog dander‐sensitized children and 20 healthy controls aged 10–18 years.^10^ Transcriptome analysis identified 321 genes that were significantly differentially expressed (DEG) between the case and control groups of those were 108 genes significantly upregulated and 213 significantly downregulated. Among the 321 DEG, cystatin‐1 (*CST1*, Cystatin‐SN) and chemokine (C‐C motif) ligand 26 (*CCL26*, Eotaxin‐3) showed the highest upregulation.

*CST1* can be found in biological fluids and leads to reversible and competitive inhibition of cysteine proteinases known as cathepsins.^11^ Furthermore, cystatins have potent immunomodulatory functions by inducing the synthesis of *TNF‐α* and *IL‐10*.^12^ Several studies of airway epithelial, bronchial and nasal tissue showed repeatedly that *CST1* is linked to asthma development outlining it as a potential biomarker and a candidate therapeutic target in allergic patients.^10^, ^13^, ^14^, ^15^ A recent study by Wang et al.^16^ identified *CST1* as a potential biomarker for asthma by analyzing 6 datasets of asthmatic patients. Upregulation of *CST1* in bronchial or nasal epithelium of asthmatic patients, highlights the role of *CST1* in the pathophysiology of asthma.^17^, ^18^

*CCL26* is a selective agonist for CC chemokine receptor 3 (CCR3) and attracts CCR‐3‐carring cells like eosinophils,^19^ basophils^20^ and Th2 lymphocytes,^21^ the most prominent cell types in allergic asthma.^22^ In line with this function, *CCL26* has been linked to asthma in several previous studies.^23^, ^24^, ^25^ Even though several studies showed an association between increased levels of *CST1* and *CCL26* and the development of asthma, it was to our knowledge so far not possible to understand the contribution of *CST1* and *CCL26* in the disease development. Obtaining more knowledge about the mechanistic insights of those genes, it might be possible to identify whether *CST1* and *CCL26* can be potential drug targets for asthma treatment. Therefore, the aim was to investigate the functional mechanisms of *CST1* and *CCL26* in an alveolar basal epithelial cell line to understand their function in asthma pathogenesis better.

## 2 MATERIAL AND METHODS

### 2.1 Cell culture

The human alveolar basal epithelial cells A549 (nonsmall lung cancer, CCL‐185™) were obtained from ATCC and cultured in complete growth medium F12/K (Thermofisher Scientific), with 10% fetal bovine serum (FBS, Nordic Biolabs) 100 units/mL penicillin and 100 μg/mL streptomycin (Corning). The cells were incubated at 37°C with 5% CO_2_. Cells were routinely checked for mycoplasma contamination using a commercially available kit (Sigma).

### 2.2 Plasmids and transfections

Expression plasmids containing the human *CST1* and *CCL26* as well as the empty vector pCMV6‐entry as control were obtained from OriGene Technologies Inc. (Table 1). A549 cells were transfected with 1 µg plasmid DNA containing *CST1* (*CST1* overexpression) or *CCL26 (CCL26* overexpression) separately in 24‐well plates using Lipofectamine® 3000 (Thermofisher Scientific, USA), according to the manufacturer's instruction. The cells were harvested for subsequent experiments 48 h following transfection. All experiments were performed in biological quadruplicates.

**Table 1:** Plasmids obtained from OriGene.

	Catalog number	RefSeqID	Cloning vector	Number of biological replicates
**Cystatin**	RC202896	NM_001898	pCMV6‐Entry	Four samples
**Eotaxin 3**	RC212044	NM_006072	pCMV6‐Entry	Four samples
**pCMV6‐Entry (control samples)**	PS100001			Four samples

### 2.3 Reverse transcription and real‐time PCR (rt‐qPCR)

Total RNA was extracted with the Qiagen RNeasy Micro kit (Hilden, Germany) 48 h after transfection and the concentrations were measured using Qubit^TM^ Flex Fluorometer (Thermofisher Scientific). RIN values were obtained from Qsep100 Bio‐Fragment Analyzers (Bioptic, Inc.) and ranged between 8.9 and 10. Overexpression was confirmed using rt‐qPCR (Figure S1). cDNA was synthesized using SuperScript III First‐Strand Synthesis SuperMix (Thermofisher Scientific), according to the manufacturer's protocol. For rt‐qPCR, 1 µL of cDNA was used in a 20 µL reaction volume, TaqMan gene expression Master Mix and pre‐developed TaqMan gene expression assays (Thermofisher Scientific). All samples were run in triplicates according to the manufacturer's instructions. Each reaction was normalized to the *GAPDH* and *PPIA* content separately and the ΔΔCT method^26^ was used to determine the fold induction over cells transfected with the empty vector (control samples).

### 2.4 Transcriptome library preparation and sequencing

In total, 12 samples were included (4 biological replicates of *CST1*, 4 biological replicates of *CCL26*, 4 biological replicates of pCMV6‐Entry). Library preparation and sequencing were performed by the National Genomics Infrastructure (NGI) in Stockholm, Sweden. The libraries were prepared with the Illumina® TruSeq Stranded mRNA library preparation kit with 330 ng total RNA of each sample by Agilent Bravo workstation, as based on the NGI sample recommendations. The average library length for *CST1* was equal to 362 bp, for *CCL26* equal to 400 bp and for pCMV6‐entry equal to 395 bp. Sequencing was performed on an Illumina^Ⓡ^ NovaSeq. 6000 platform using the NovaSeq. 6000 S4 Reagent Kit (35 cycles) (Illumina).

### 2.5 Processing of RNA sequencing reads and calculation of differential gene expression

Quality of raw reads was evaluated using FastQC (v0.11.1).^27^ Reads were trimmed to remove low quality bases using Trim Galore (v0.6.1).^28^ Expression estimates were obtained by kallisto quant^29^ using human reference transcriptome from Ensembl release 107.^30^ Per‐transcript count estimates were summarized at the gene level using tximport (v.1.28.0), as described in Soneson et al 2015^31^ and normalized using variance stabilizing transformation from DESeq. 2 (v.1.40.2)^32^ for plotting. Differential expression was tested using glmQLFTest^33^ from edgeR (v.3.42.4)^34^ using trimmed mean of M values (TMM) scaled expression estimates. Log_2_ transformed fold change (FC), *p*‐value, FDR^35^ were calculated for each gene. We considered |log_2_FC | ≥ 2 and FDR < 0.01 as the cutoff values to identify differentially expressed genes.

### 2.6 Pathway enrichment

Gene set enrichment analyses for biological processes were performed using clusterprofiler (v.4.8.3)^36^ and the network analysis was completed with ShinyGO (v0.77).^37^

### 2.7 Proteomic analysis

The protein expression of A549 cells overexpressing *CST1* and *CCL26* separately as well as the empty vector was analyzed with the antibody‐mediated proximity extension–based assay OLINK (Proteomics, Uppsala, Sweden) using the Target 96 inflammation panel. RIPA lysis buffer (Merck) supplemented with proteases inhibitors (Roche) was used to collect the cell lysate 48 h after transfection. Lysis buffer only was included as negative control. The measurements were conducted in duplicates at the affinity proteomics‐Stockholm facility as previously described.^38^

### 2.8 Processing of proteomic data

The protein expression levels are presented as normalized protein expression (NPX) values in a log_2_‐scale. If 15% of the samples from one protein had values below the limit of detection (LOD), the proteins were excluded from the analysis (Table S3). The difference between the protein expression levels of cells overexpressing the gene of interest or the control vector, delta‐NPX, were compared using two‐tailed *t*‐test in Microsoft Excel. Delta‐NPX is presented as log_2_FC and proteins with |log_2_FC | ≥ 1 and *p* < .05 were considered as differentially expressed proteins. ClustVis (v.1.0)^39^ was used to generate the heatmaps of the expression data.

## 3 RESULTS

**Figure 1:**
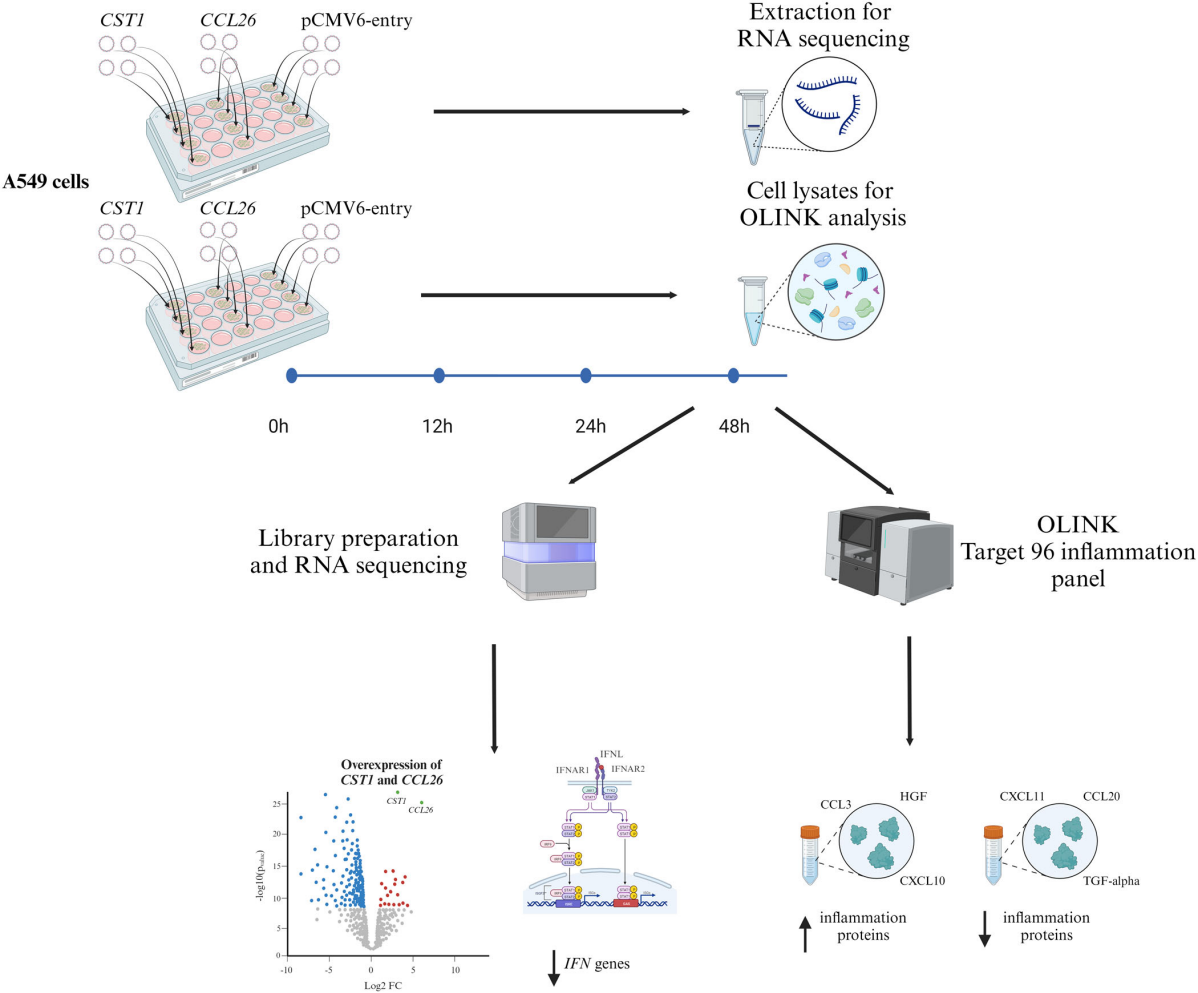
Flow chart of the study. A549 cells were separately transfected with the expression plasmids containing human CST1 and CCL26 as well as the empty vector pCMV6‐entry as control, obtained from Origene Technologies Inc. In total, 12 samples were included (4 biological replicates of CST1, 4 biological replicates of CCL26, 4 biological replicates of pCMV6‐Entry). On the one hand, RNA was extracted with the Qiagen RNeasy Micro kit 48 h after transfection and on the other hand, the cell lysates were collected with RIPA lysis buffer. Transcriptomic analysis was conducted via RNA sequencing and proteomic analysis via the Target 96 inflammation panel from OLINK. The transcriptomic analysis was conducted in biological quadruplicates and the proteomic analysis in biological duplicates. The results identified a downregulation of type I and III interferon genes as well as differentially expression of inflammatory proteins. Created with BioRender.

A549 cells were transfected with either *CST1* or *CCL26* to investigate the functional mechanism of these genes (Figure 1).

**Figure 2:**
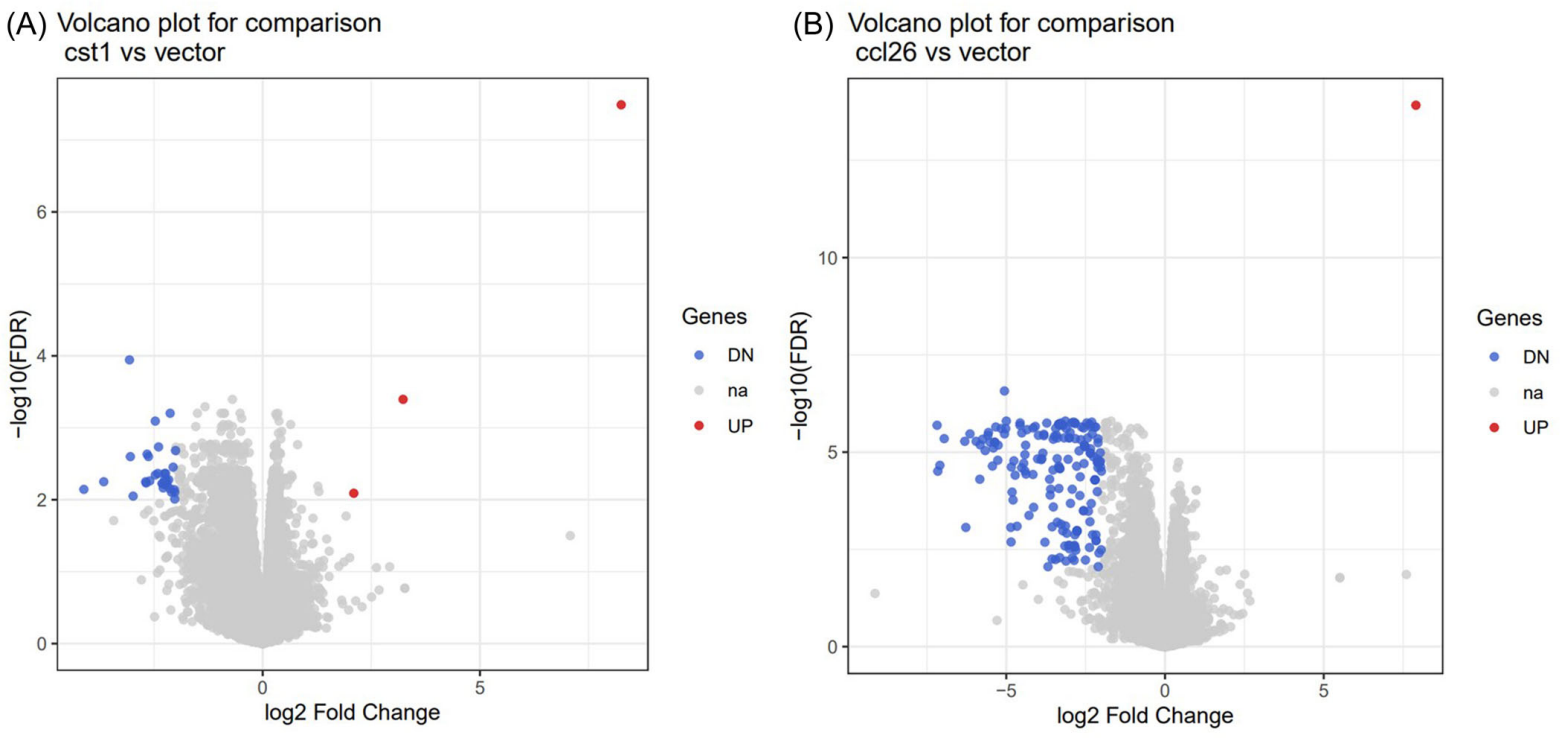
Volcano plot of the differentially expressed genes after the overexpression of (A) CST1 and (B) CCL26, separately. CST1 and CCL26 are in the upper right corner of the respective volcano plot. Gray: nonsignificantly expressed genes, blue: significantly downregulated genes, red: significantly upregulated genes with a |log_2_FC | ≥ 2. FDR, false discovery rate.

The overexpression was confirmed with rt‐qPCR (Figure S1) and downstream effects were evaluated with RNAseq. edgeR analysis was performed on the RNAseq data to obtain the differentially expressed gene profiles ( | log_2_FC | ≥ 2, FDR < 0.01). The overexpression of *CST1* in A549 cells resulted in a total of 27 differentially expressed protein coding genes. Out of the 27 DEG, 1 was upregulated and 26 downregulated (Figure 2, Table 2, Figure S2A, Table S1). An overexpression of *CCL26* resulted in a total of 137 differentially expressed protein coding genes of those no gene was upregulated and 137 were downregulated (Figure 2, Table 2, Figure S2B, Table S2).

**Table 2:** Differentially expressed genes after overexpression of CST1 and CCL26, separately.

Differentially expressed genes	After *CST1* overexpression	After *CCL26* overexpression
Upregulated genes	1	0
Downregulated genes	26	137

Differentially expressed genes	Upregulated genes	Downregulated genes
After *CST1* overexpression	*CST2*	*RUFY4, UBA7, IFNB1, IFNL2, SMTNL1, IFNL1, HLA‐F, IFIT2, IDO1, XAF1, RSAD2, HSH2D, ETV7, BST2, SECTM1, HLA‐B, GMPR, IFI27, LAMP3, GH1, OAS2, APOL1, IFITM1, OASL, CMPK2, PARP10*
After *CCL26* overexpression		*IFI27, RTP4, SLC15A3, RSAD2, IFI44L, RUFY4, IFIT2, IFNB1, IDO1, OAS2, CMPK2, IFIT1, IFIT3, BST2, SMTNL1, OASL, ISG15, XAF1, IFI44, IFITM1, GBP1, GH1, IFI6, MX1, UBA7, IFNL3, CXCL11, HSH2D, IFNL2, APOL3, IFNL1, HLA‐F, CXCL10, MX2, BATF2, LAMP3, LGALS9, HLA‐B, TNFSF10, IFIH1, IRF7, PARP10, GBP4, RIGI, CASP1, SAMD9L, THEMIS2, TRANK1, CCL5, TRIM22, APOL1, DHX58, PSMB8, HELZ2, SH2D1B, TLR3, SECTM1, CFB, EPSTI1, PLEKHA4, IFI16, ETV7, IFI35, HERC5, SERPING1, USP18, DDX60, PSMB9, IFITM3, GMPR, RASGRP3, SAMD9, PPP4R4, PARP9, CYP2J2, NLRC5, TNFSF13B, SP110, UBE2L6, IL1RN, OAS3, CLEC7A, RAET1L, PLAAT4, LMO2, OAS1, RGS22, SOCS1, PARP12, GIMAP2, C4A, DDO, SAA2, CACNA1I, STAT2, SHFL, DDX60L, PDCD1LG2, ODF3B, TYMP, TXNIP, APOL6, DTX3L, TAP1, PARP14, NT5C3A, SAMHD1, GBP3, ARC, HERC6, TRIM21, SP140L, ZMYND15, PSORS1C1, C1R, STAT1, PPM1K, PLA1A, TRIM14, LGALS3BP, CD274, CD68, PLSCR1, PML, ISG20, TAP2, MAP2, LAP3, HLA‐C, APOL2, KLK10, RET, TNFSF14, TREX1, REC8, BHLHE41, CEACAM1*

Gene ontology enrichment analysis was performed for all downregulated genes (log_2_FC ≤ −2, FDR < 0.01) due to the overexpression of *CST1* and *CCL26*, separately, based on biological processes.

**Figure 3:**
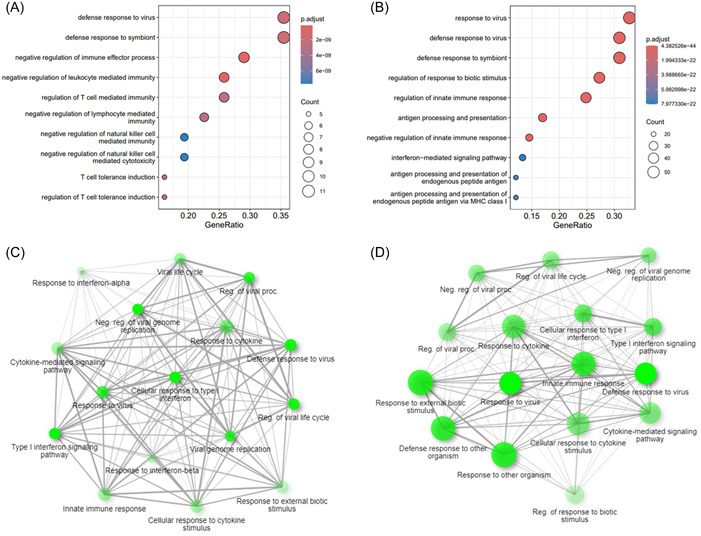
Gene ontology enrichment analysis for the downregulated genes due to (A) CST1 overexpression (*n*_downregulated_ = 26) and (B) CCL26 overexpression (n_downregulated_ = 137). The x‐axis represents the GeneRatio, number of gene count indicated by the dot size, adjusted p‐value indicated by color (red = high significant, blue = low significant); the *y*‐axis represents the gene ontology biological processes. Network analysis of the relationship between the enriched pathways for the downregulated genes due to (C) CST1 overexpression (n_downregulated_ = 26) and (D) CCL26 overexpression (n_downregulated_ = 137). Two nodes are connected if they share 20% (default) or more genes. Darker nodes = more significantly enriched gene sets, bigger nodes = larger gene sets, thicker edges = more overlapped genes.

**Figure 4:**
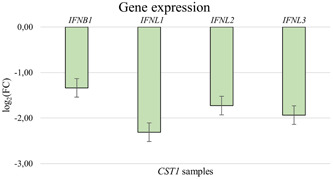
Confirmation of the RNAseq results via rt‐qPCR for the CST1 samples.

**Figure 5:**
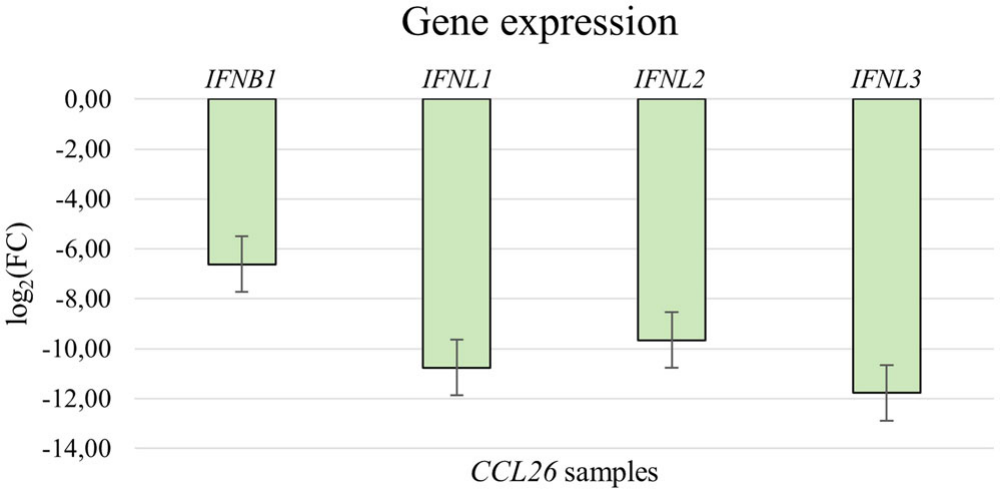
Confirmation of the RNAseq results via rt‐qPCR for the CCL26 samples.

For the protein coding downregulated genes following *CST1* overexpression (*n* = 26), defense response to virus, defense response to symbiont, negative regulation of immune effector process were the top three biological processes (Figure 3A). The downregulated protein coding genes due to *CCL26* overexpression (*n* = 137) showed as top 3 biological processes response to virus, defense response to virus and defense response to symbiont (Figure 3B). The RNAseq results were confirmed via rt‐qPCR and showed a downregulation of *IFNB1* as well as *IFNL1‐3* for the samples overexpressing *CST1* (Figure 4) as well as *CCL26* (Figure 5). The network analyses of the downregulated genes for both *CST1* and *CCL26* overexpression models are shown in Figure 3C,D.

**Figure 6:**
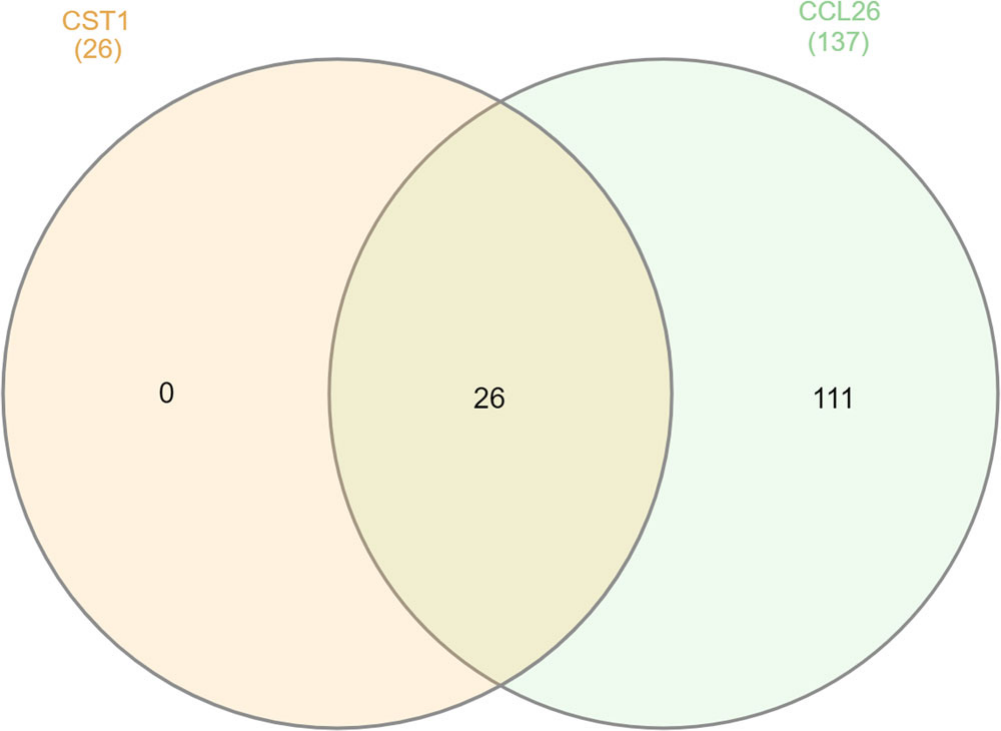
Venn diagram of differentially expressed downregulated protein coding genes after either CST1 or CCL26 overexpression.

All downregulated genes after overexpression of *CST1* overlap with the downregulated genes after *CCL26* overexpression (Figure 6).

Protein levels of the A549 cells overexpressing *CST1* or *CCL26* were analyzed with the antibody‐mediated proximity extension–based assay OLINK using the Target 96 inflammation panel. In A549 cells overexpressing *CST1*, only CCL3 was differentially higher expressed with a log_2_FC of 1.99, and no protein was differentially lower expressed. Among the samples overexpressing *CCL26*, in total 5 proteins were differentially expressed, HGF was higher expressed with a log_2_FC of 1.10 and CXCL11, CCL20, CCL3 and CXCL10 were lower expressed with a log_2_FC of −1.73, −1.64, −1.12, and −1.07, respectively (*p* < .05, Table 3, Tables S4, S5, Figure S3).

**Table 3:** Top 10 higher and lower expressed proteins in A549 cells overexpressing CST1 and CCL26 separately.

A549 cells overexpressing CST1	Top 10 proteins with increased expression		A549 cells overexpressing CST1	Top 10 proteins with decreased expression	
**Proteins**	**log**_**2**_ **FC**	***p*‐value**	**Proteins**	**log**_**2**_ **FC**	***p*‐value**
CCL3	1.99	**.004**	CASP‐8	−0.84	.352
CXCL10	0.74	**.004**	TGF‐alpha	−0.51	**.025**
MCP‐1	0.54	.065	IL18	−0.50	.055
CCL20	0.54	.058	ADA	−0.40	.081
IL8	0.53	.055	ST1A1	−0.28	.280
CDCP1	0.52	.069	CSF‐1	−0.13	.335
IL‐1 alpha	0.50	.135	SIRT2	−0.08	.709
CXCL1	0.48	.091	HGF	−0.04	.898
TNFRSF9	0.47	**.049**	4E‐BP1	−0.04	.765
IL6	0.47	.089	VEGFA	−0.04	.589

A549 cells overexpressing CCL26	Top 10 proteins with increased expression		A549 cells overexpressing CCL26	Top 10 proteins with decreased expression	
**Proteins**	**log**_**2**_ **FC**	***p*‐value**	**Proteins**	**log**_**2**_ **FC**	***p*‐value**
HGF	1.10	**.038**	CXCL11	−1.73	**.013**
LAP TGF‐beta‐1	0.41	.127	CCL20	−1.64	**.006**
IL‐22 RA1	0.37	.105	CCL3	−1.12	**.031**
AXIN1	0.36	.107	CXCL10	−1.07	**.004**
SIRT2	0.31	.228	CASP‐8	−0.97	.301
MMP‐1	0.24	.058	TGF‐alpha	−0.87	**.009**
IL‐10RB	0.23	.153	CD40	−0.66	**.045**
STAMBP	0.19	.328	PD‐L1	−0.66	**.039**
TWEAK	0.18	.192	IL‐1 alpha	−0.64	.103
SCF	0.18	.128	Flt3L	−0.58	**.020**

## 4 DISCUSSION

Increased expression of *CST1* and *CCL26* has been previously shown in nasal epithelium of children with asthma and allergy,^10^ however, the functional mechanism of how it contributes to asthma development is so far not well understood. Therefore, our study examined the transcriptomics and inflammatory proteins due to the overexpression of *CST1* and *CCL26* separately in the alveolar basal epithelial cell line A549. Significant downregulation of genes involved in type I and III interferon signaling pathway and several interferon‐stimulated genes, especially due to the overexpression of *CCL26* was seen. Also on protein level, the overexpression of *CCL26* induced slightly more differentially expressed proteins. Due to the downregulation of interferon type I and III genes, we hypothesize that the overexpression of *CST1* and *CCL26* results in a more severe airway inflammation and disease progression.

In our study, the transcriptomic profile after overexpression of *CST1* induced an upregulation of one (*CST2*) and a downregulation of 26 protein coding genes. *CST2* (Cystatin‐SA), a thiol protease inhibitor, is the significantly upregulated gene and, like *CST1* protects against allergens, viral and bacterial proteases and is highly expressed in salivary glands.^40^, ^41^ Both *CST1* and *CST2* consist of 141 amino acids and include cysteine protease inhibitor, proteinase inhibitor I25A and I25B, type 2 and phytocystatins and *CST1* also a Myb DNA‐binding domain^42^, ^43^ and it could be previously seen that the genes are closely linked to each other.^44^ A recent publication from Nocera et al.^45^ identified overexpression of *CST2* in patients with chronic rhinosinusitis with nasal polyp (CRSwNP) compared to controls.

The overexpression of *CCL26* in the alveolar basal epithelial cell line A549 caused no upregulation of any gene.

An enrichment of genes involved in interferon signaling, including interferon‐stimulated genes was seen to be downregulated in the alveolar basal epithelial cells after overexpression of *CST1*. It resulted among others in the downregulation of the type I and III interferons *IFNB1* and *IFNL1* (*IL‐29*) *and IFNL2 (IL‐28A)*. The function of interferons is generally the induction of antiviral enzymes and interferon‐stimulated genes.^46^ Several interferon‐stimulated genes linked to viral replication were downregulated in the *CST1* overexpressing cells. Among those, *IFITM1* (interferon‐induced transmembrane protein 1) that inhibits the viral entry via the endocytic vesicle membrane which fuses with incoming viruses.^47^ Moreover, *IFIT2*, inhibits viral protein production during viral mRNA translation.^48^ The viral replication of different RNA and DNA viruses is suppressed by *RSAD2* (viperin) a radical SAM (S‐adenosyl‐l‐methionine) enzyme, due to its interaction with cellular and viral proteins.^49^ *OAS2* (oligoadenylate synthetases) degrades viral genomes^50^ and *BST2* (tetherin), a transmembrane protein, prevents at the last stage of the viral cycle, the release of virions.^51^ Furthermore, the HLA class I (major histocompatibility complex, class I, human leukocyte antigen, MHC class I) genes *HLA‐B* and *‐F* were downregulated after the overexpression of *CST1*. MHC class I genes present antigens to cytotoxic CD8^+^ T cells, which activates them to eliminate infectious cells and to produce inflammatory cytokines like IFNγ.^52^ In contrast to our results, previous findings showed increased *HLA‐F* surface expression in severe asthmatic patients.^53^ However, a downregulation of MHC class I could be observed in human blood cell lines as well as the alveolar basal epithelial cell line A549 after the infection with influenza A and B,^54^ nevertheless, our cells overexpressed *CST1* and had no virus infection. We can speculate that an overexpression of *CST1* mimics a virus infection and causes therefore a downregulation of *HLA‐F*.

It is known that rhinovirus especially causes wheezing symptoms in preschool children and infection can increase the risk for asthma.^55^ Our findings of the downregulation of genes involved in viral defense mechanism following *CST1* overexpression, suggest that higher levels of *CST1* may facilitate viral entry. The consequence may be an increased rhinovirus load that might lead to the development of asthma later in life. As a rhinovirus infection cause an inflammation of the upper and lower airways, it is a potential trigger in the development of asthma.^56^

Genes downregulated due to *CST1* or *CCL26* overexpression overlap (Figure 6). Comparing the significantly downregulated genes after *CCL26* and *CST1* overexpression, almost all of these 137 genes were significantly downregulated also due to *CST1* overexpression, however with a log_2_FC below our cut‐off (data not shown). That might indicate that *CST1* as well as *CCL26* have similar effects or are regulated by each other. As *CCL26* attracts eosinophils, this interaction is supported by previous studies showing that *CST1* progresses the migration of eosinophils and might play a role in the development of chronic rhinosinusitis.^57^, ^58^

In addition to the downregulated genes already discussed, the interferon type III (*IFNL3* (*IL‐28B*)) gene as well as 110 more genes, were downregulated due to *CCL26* overexpression. Genes with a function within interferon signaling or similar are discussed in the following. *RIG‐I* (*DDX58*), a cytosolic sensor of viral RNA acts via the adapters MAVS and STING and activates the type I interferon pathway, also via *IRF7*.^59^ The interferon‐repressed genes *STAT1* and *STAT2* are transcription factors in the interferon signaling pathways. Via the JAK/STAT pathway, the transcription factor complex gets activated, which consists of SH2‐phosphotyrosine‐mediated heterodimer *STAT1* and *STAT2* as well as *IRF9*.^60^ *USP18* (Ubiquitin‐specific protease) regulates negatively the *IFN* induction^61^ and *ISG20*, a 3′−5‘ exonuclease, degrades viral genes and genome.^62^ Furthermore, several studies showed that *MX1* suppresses influenza A virus infections,^63^, ^64^ *MX2* HIV‐1 infections^65^, ^66^ and *IFI6* the replication of flavivirus.^67^ *IFITM3* inhibits the viral entry like previously mentioned *IFITM1* and *IFI16* is a sensor for viral DNA and RNA.^47^, ^68^ The poly‐ADP ribose polymerase (*PARP*) suppresses viral replication by building complexes with the ribosome.^69^ The genes downregulated due to *CCL26* overexpression are again linked to viral entry as well as inflammation. An increase viral load due to an enlarged viral entry is known to increase the risk for asthma development later in life.^55^ Inflammation pathways like JAK/STAT, involved in the regulation of Th2 cells^70^ are moreover a potential target for the treatment of allergic diseases.^71^

Our results indicate that the overexpression of *CCL26* as well as *CST1* causes a downregulation of several antiviral response genes which has previously been seen in patients with asthma and allergy. Hwang et al.^72^ investigated sinonasal epithelial cells of patients with chronic rhinosinusitis with and without nasal polyps comparing it to healthy controls. A decreased expression of *IFNB*, *IFNL1*, *IFNL2*, *OAS*, *MX1* and *RSAD2* was seen in both RNA and protein levels in sinonasal mucosa with inflammation compared to normal mucosal tissue. Although the findings are from nasal epithelium, they are in line with our hypothesis that the overexpression of *CCL26* causes a more severe asthma phenotype, based on previous findings in nasal epithelium.^10^ Furthermore, a reduced induction of *IFNL* genes could also be seen in asthmatic primary bronchial cells and alveolar macrophages after rhinovirus infection^73^ as well as for interferon beta.^74^

Due to the findings of this current and previous studies, we hypothesize that the overexpression of *CST1* and *CCL26*, lead to a downregulation of interferon type I and III genes, which in turn may cause a more severe airway inflammation and progression of the disease. Our findings are in line with the theory proposed by Holt et al.,^75^ showing that lower interferon levels at birth caused a more severe infection of the lower respiratory tract later in life. That might repose that the viral infections cannot be well controlled as genes involved in the inhibition of the viral replication cycle are not sufficiently expressed, and it results therefore in a deficient immune response and a higher disease susceptibility.

We hypothesize that heavily increased expression of *CCL26* causes a more severe phenotype of asthma, especially allergic asthma, as *CCL26* is also an agonist for CCR‐3‐carrying cells, like eosinophils^19^ and Th2 lymphocytes,^21^ the most prominent cell type in allergic asthma. Asthma is often paired with allergic sensitization^76^ and the most prevalent type of asthma in children.^3^ Due to the damage of the airway mucosa upon viral infection, patients are more susceptible to allergens.^77^

Increased expression of type I interferon could also be seen in nasal epithelium of children compared to adults infected with SARS‐CoV‐2,^78^ which indicates different mechanism between adults and children. However, an increase of *IFNL* in a cohort of wheezing children aged 5–18 years was associated with asthma exacerbation.^79^ Nevertheless, studies in mice models^80^ and human peripheral blood mononuclear cells^81^, ^82^ showed that an administration of type I and III interferons decreased inflammation in the airways due to a decreased IL‐13 secretion and inhibition of Th2 cells. These data indicate that the role of interferon levels in asthma development has yet to be fully elucidated.

Different findings regarding an up‐ or downregulation of type I and III interferons in association with asthma have been identified in several studies (Table 4). Due to the fact that several genes have been associated as asthma susceptibility genes,^6^ it might be speculated that increased or decreased levels of type I and III interferon genes as well as interferon‐stimulated genes are linked to the enormous overexpression of a specific gene, in our study *CST1* and *CCL26*. Therefore, further studies investigating asthma associated genes and their downstream effects are warranted. It would be hereby possible to identify the function of more asthma susceptibility genes.

**Table 4:** Overview about findings regarding increased and decreased levels of type I and III interferons.

Downregulation of type I and III interferons	Study population	Results
Hwang et al. 2019^72^	Sinonasal epithelial cells of patients with chronic rhinosinusitis	Downregulation *of IFNB, IFNL1, IFNL2, OAS, Mx1 and RSAD2* on gene and protein level in patients with chronic rhinosinusitis
Contoli et al. 2006^73^	Primary bronchial epithelial cells of asthmatic patients	Reduced induction of *IFNL* after rhinovirus infection
Wark et al. 2005^74^	Bronchial epithelial cells of patients with moderately severe asthma	Lower levels of *IFNB* after rhinovirus infection

Upregulation of type I and III interferons	Study population	Results
Miller et al. 2012^79^	Nasal epithelial cells of wheezing children	Increased *IFNL* expression with or without HRV infection in wheezing children
Bullens et al. 2008^83^	Airways cells from sputum of asthmatic adults and asthmatic school‐aged children	Increased *IFNL* expression in asthmatic patients
da Silva et al. 2017^84^	Sputum samples of adult asthmatic patients	Increased expression of *IFNB*, *IFNL1* in patients with neutrophilic asthma

On the protein level, the OLINK analysis showed that the overexpression of *CCL26* induced more differentially expressed proteins than *CST1*, which is consistent with the transcriptomic data, strengthening our hypothesis that especially *CCL26*, causes a more severe airway inflammation, particularly in allergic asthma.

Due to the overexpression of *CCL26*, the chemokines CXCL10, CXCL11, CCL20 and CCL3 were significantly downregulated, supporting hereby the findings of downregulation for CXCL10, CXCL11 and CCL20 on RNA‐level (log_2_FC < −1.6). A previous study of cord blood found associations between higher CXCL10 levels in infancy and asthma later in life, contradictory to our findings, but decreased levels of CXCL11 that were associated with allergic sensitization later in life, in line with our study.^85^ In plasma extracellular vesicles of allergic patients, CCL20 was among the downregulated biomarkers^86^ what is in line with our results. Nevertheless, higher CCL3 concentration in serum could be observed in patients with allergic rhinitis,^87^ only seen for the samples overexpressing *CST1*.

Though, the previous findings were observed in cord blood and serum, to the best of our knowledge, in airway tissue the information on their expression levels is limited.

A previous study in A549 cells investigated the overexpression of the Toll‐like receptor (TLR), that has an important role in the innate immune system, and observed a decreased expression of among others CXCL10 and CCL20 in the cell supernatant due to the overexpression of TLR10,^88^ similar to our findings in cell lysate. Furthermore, a meta‐analysis of nasal epithelium from children between 6 and 16 years of age, demonstrated decreased expression of CXCL11 and CXCL10 in patients with obesity‐related asthma^89^ and is also in line with our hypothesis that the overexpression of *CCL26* causes a more severe asthma phenotype, based on previous findings in nasal epithelium.^10^

In a study of A549 cells infected with respiratory syncytial virus (RSV), higher concentrations of CXCL10 and CCL3 were identified in the cell supernatant 24 h after RSV infection.^90^ Our study observed a downregulation of CXCL10 and CCL3 due to the overexpression of *CCL26*, but the overexpression of *CST1* induced a significant upregulation of CCL3. The cells in the current study were only overexpressed with *CST1* and *CCL26* separately, but not infected with any virus and intracellular proteins were measured.

HGF with its function to decrease airway inflammation and airway remodeling,^91^ was increased expressed in A549 cells overexpressing *CCL26*, and is against our hypothesis that an overexpression *CCL26* causes a more severe asthma phenotype, nevertheless it was slightly lower expressed due to the overexpression of *CST1*, but not significant. In the current study, four proteins (CXCL11, CCL20, CCL3, CXCL10) that were downregulated due to the overexpression of *CCL26*, are slightly upregulated due to the overexpression of *CST1*, however, only significantly different for CCL3. Overall, the findings pointing to the direction that the overexpression of both *CST1* as well as to a stronger extend *CCL26* cause an alteration of genes involved in type I and III interferon response as well as inflammatory proteins. Also seen on mRNA level as all downregulated genes due to the overexpression of *CST1* overlap with the downregulated genes after *CCL26* overexpression.

### 4.1 Clinical implications and future perspectives

Overall, our findings increase the knowledge of the underlying mechanism of the overexpression of *CST1* and *CCL26*, namely, decreasing the expression levels of interferons, which are important components of the viral defense. Wheeze is a common symptom in children, most commonly induced due to a rhinovirus infection and associated with asthma development.^56^ Our results may indicate that the overexpression of *CST1* and *CCL26* causes a more severe airway inflammation, particularly in allergic asthma, as well as an impaired immune system. Therefore, *CST1* and *CCL26* might be considered as potential drug targets for asthma treatment. A direct downregulation of *CST1* and *CCL26*, could possibly increase the interferon levels, what should be investigated in future drug discovery studies. Targeted treatment can mitigate the progression of the disease as well as delay onset. This opens for the possibility to design treatments that target the cause of asthma and not just the symptoms.

### 4.2 Strength and limitations

The strength of our study is that we used the alveolar basal epithelial cell line A549, a common in vitro model for asthma research.^92^, ^93^, ^94^ However, the cells are obtained from a donor with lung adenocarcinoma, therefore, the cells differ from healthy primary cells. Nevertheless, the differentially expressed genes of both overexpression experiments, did not show an enrichment of pathways related to cancer. Furthermore, a previous study from Roberts et al.,^95^ showed that nasal and bronchial epithelial cells response similar to rhinovirus infections. The gene expression profiling of nasal and bronchial brushings from patients with airway disease and healthy controls, also showed an overlap of 98.2% DEG between the matched samples.^96^ Both studies show that the alveolar basal epithelial cell line A549, used in our study, represents a meaningful model to investigate previous findings from the nasal epithelium.

A downregulation of the type I and III interferons due to mycoplasma contamination can be excluded, as the cells were regularly tested for contamination.

We measured the protein expression levels of cells overexpressing *CST1* and *CCL26* separately via the Target 96 inflammation panel from OLINK to explore the findings on protein level, what is a strength of our study. OLINK is a highly sensitive method that allows the measurement of several proteins at the same time. In total, only 45/92 could be included in the analysis as the rest of the proteins did not pass the LOD, though this is to be expected when doing measurements from cell lysates. Nevertheless, our OLINK analyses had a small sample size with two samples in each group, which is a limitation of the study. For the transfection of A549 cells, we used the transfection delivery vehicle as transfection control to avoid experimental biases, strengthening our experimental approach. However, our study might include a confirmation bias as we have four biological replicates for the RNA sequencing samples, however, no technical replicates. Furthermore, it can be possible that we have a replicate bias for the RNA sequencing and OLINK samples as the experiments were performed in the same manner, however, not on the same day.

## 5 CONCLUSION

Our study investigated the functional effects of downstream targets following the overexpression of *CST1* and *CCL26* separately in the alveolar basal epithelial cell line A549. We found a downregulation of type I and III interferons and several interferon‐stimulated genes. It indicates that patients with upregulated *CST1* and especially *CCL26* levels also confirmed on protein level, have a deficient immune response and a higher disease susceptibility. A specific downregulation of those genes might present a drug target to increase in return the immune response.

## AUTHOR CONTRIBUTIONS

**Angela Hoyer**: Conceptualization; formal analysis; investigation; methodology; visualization; writing—original draft; writing—review and editing. **Sandip Chakraborty**: Data curation; formal analysis; software; visualization; writing—review and editing. **Ingrid Lilienthal**: Methodology; writing—review and editing. **Jon R. Konradsen**: Writing—review and editing. **Shintaro Katayama**: Writing—review and editing. **Cilla Söderhäll**: Conceptualization; funding acquisition; methodology; project administration; resources; supervision; writing—review and editing.

## CONFLICT OF INTEREST STATEMENT

The authors have no conflicts of interest to declare.

## ETHICS STATEMENT

The work in this publication is based on experiments done in commercially available human cell lines, which do not require ethical approval.

## Supporting information

Supporting information.

Supporting information.

Supporting information.

Supporting information.

## Data Availability

The data that support the findings of this study are openly available in NCBI GEO with the GEO accession number GSE229741.
