# Supplementary figures and images for: The functional role of CST1 and CCL26 in asthma development
Source: Immun Inflamm Dis. 2024 Jan 19;12(1):e1162. doi: 10.1002/iid3.1162 (PMC10797655; doi:10.1002/iid3.1162)

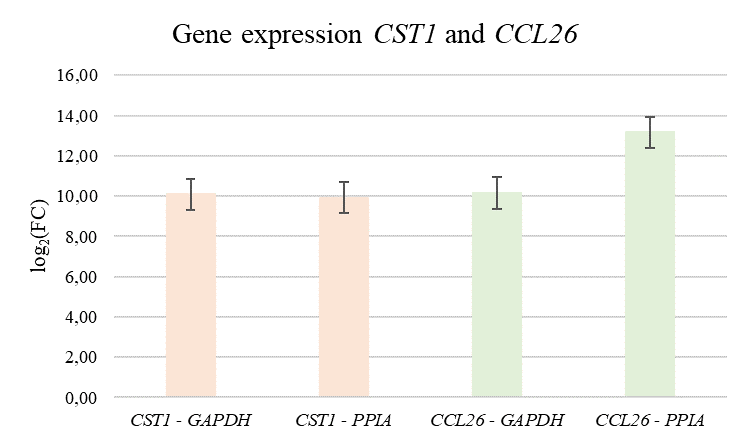

Supplement: Supplementary file 1 — Supporting information. [file IID3-12-e1162-s001.png]

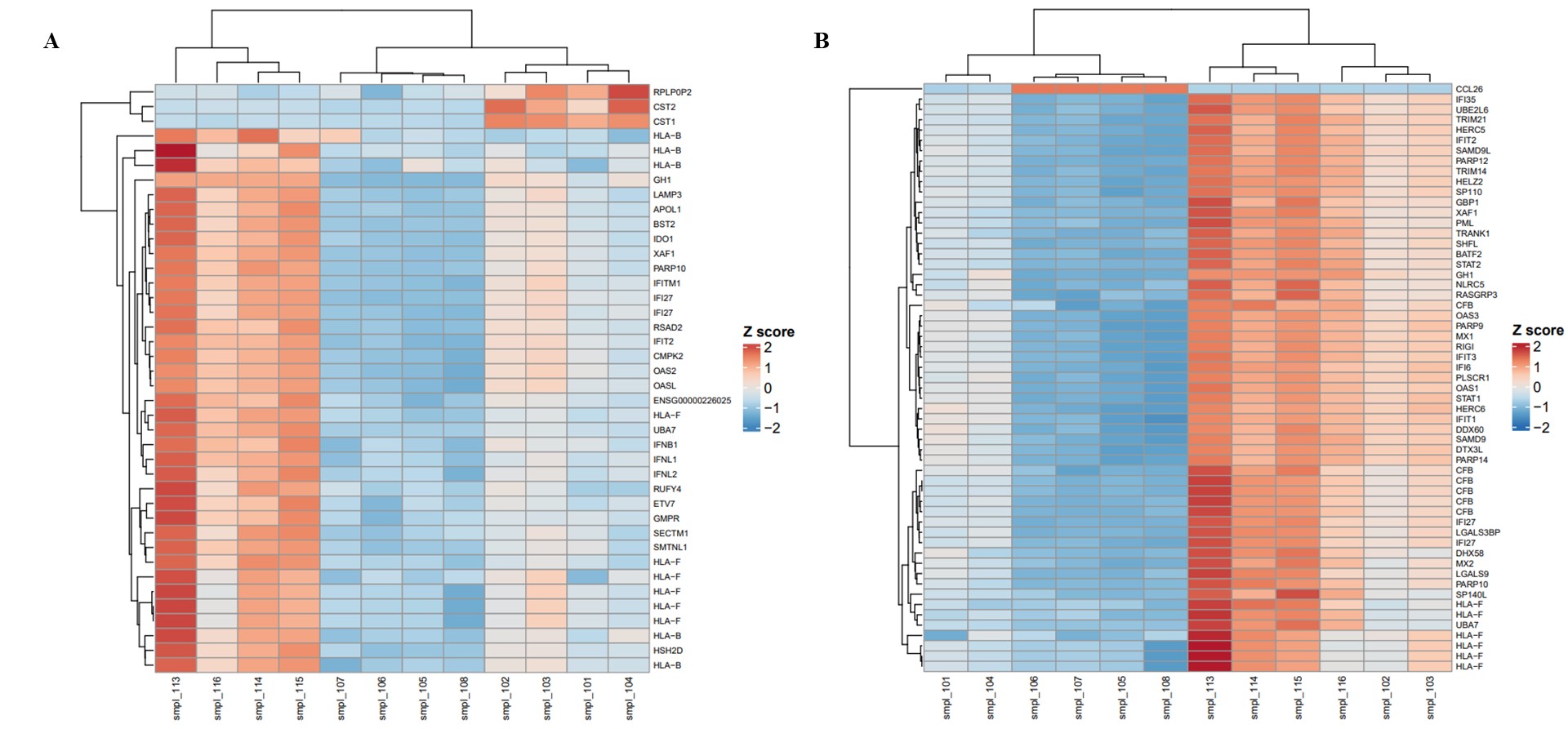

Supplement: Supplementary file 2 — Supporting information. [file IID3-12-e1162-s003.jpg]

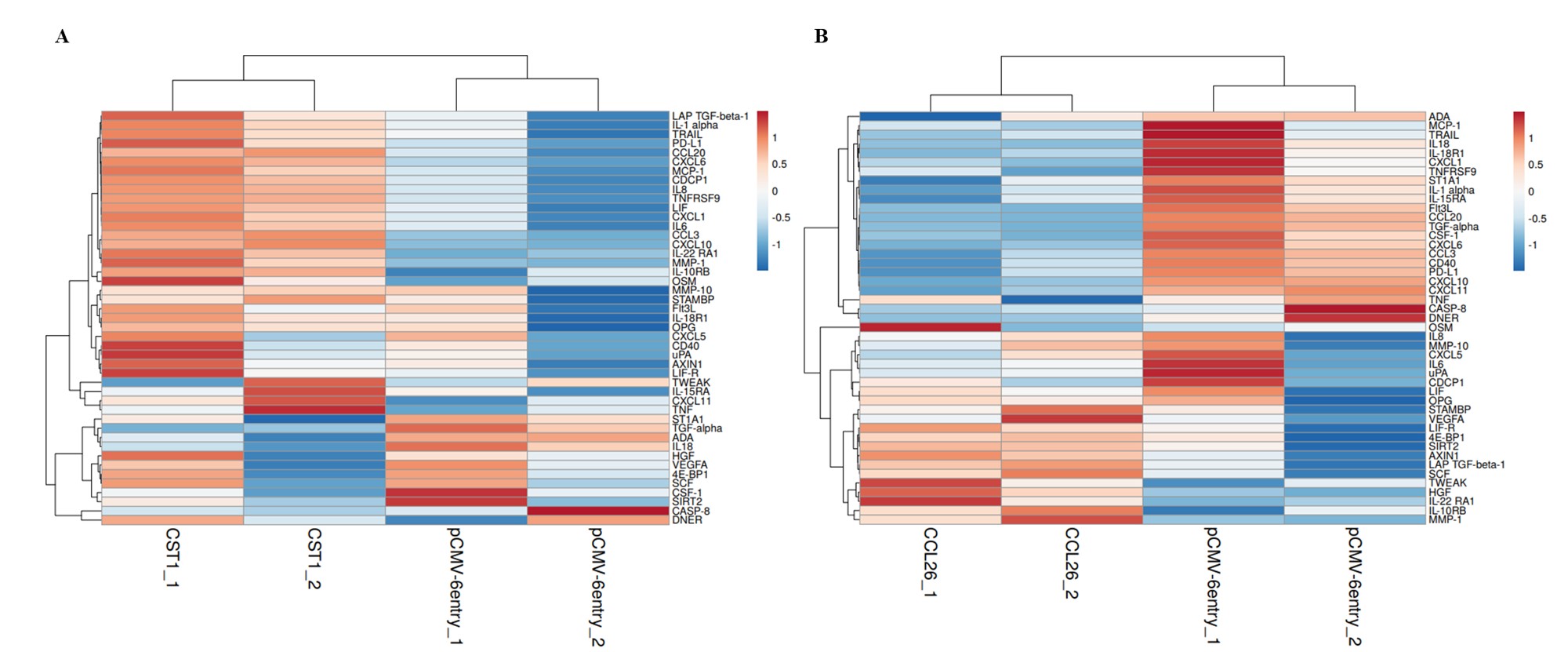

Supplement: Supplementary file 3 — Supporting information. [file IID3-12-e1162-s004.jpg]
