# Supplementary material for: The functional role of CST1 and CCL26 in asthma development
Source: Immun Inflamm Dis. 2024 Jan 19;12(1):e1162. doi: 10.1002/iid3.1162 (PMC10797655; doi:10.1002/iid3.1162)
Supplement: Supplementary file 4 — Supporting information. [file IID3-12-e1162-s002.docx]

**Supporting Material**

**Confirmation of the overexpression of *CST1* and *CCL26***

Supplementary Figure 1: Confirmation of the overexpression of CST1 and CCL26 in A549 via rt-qPCR. GAPDH and PPIA used as housekeeping genes.


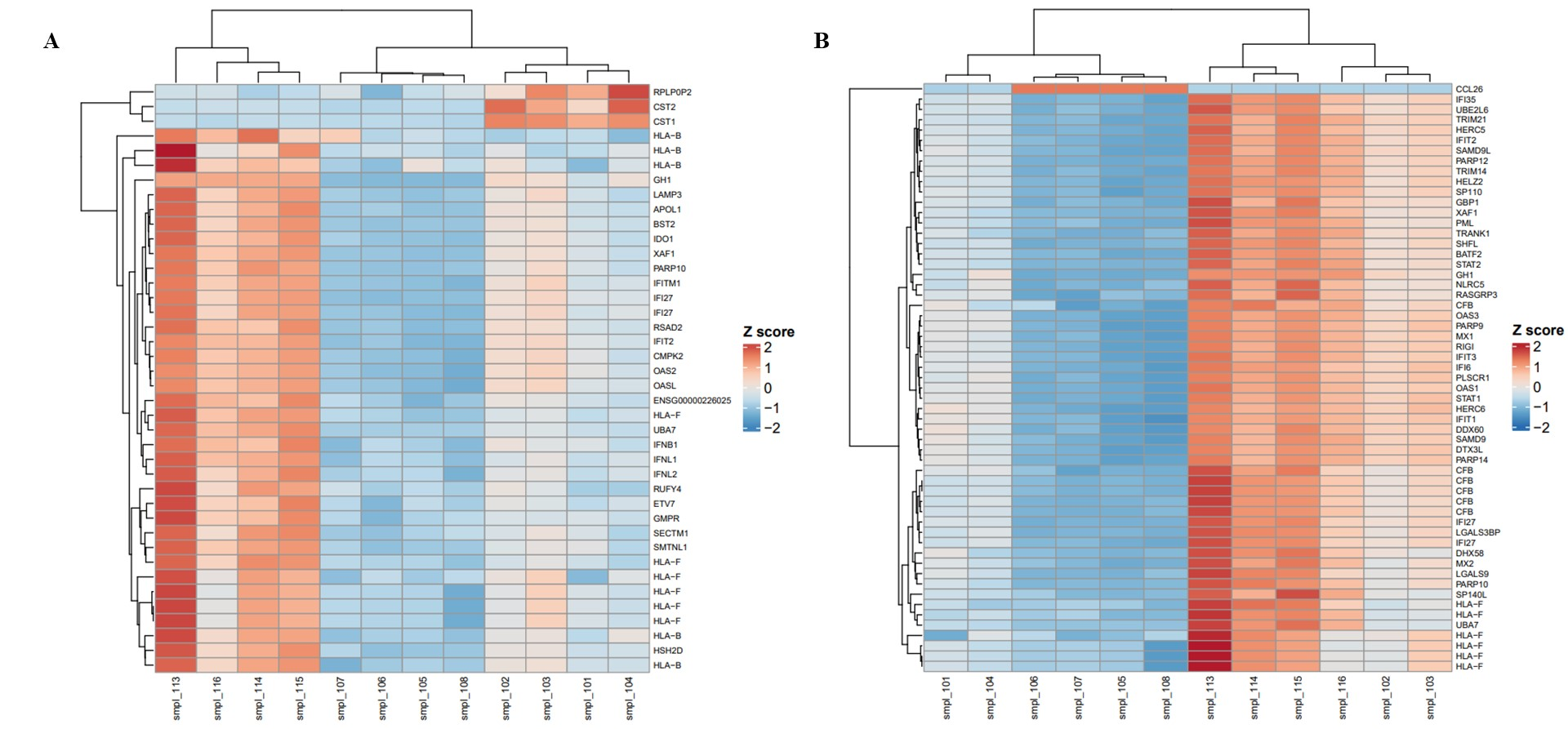


Supplementary Figure 2: Heatmap of the Top 50 genes by FDR of A549 cells overexpressing (A) CST1 and (B) CCL26 versus the control vector pCMV-6entry. smpl_101 = cst1, smpl_102 = cst1, smpl_103 = cst1, smpl_104 = cst1, smpl_105 = ccl26, smpl_106 = ccl26, smpl_107 = ccl26, smpl_108 = ccl26, smpl_113= pCMV6-Entry, smpl_114 = pCMV6-Entry, smpl_115= pCMV6-Entry, smpl_116 = pCMV6-Entry. FDR = false discovery rate.


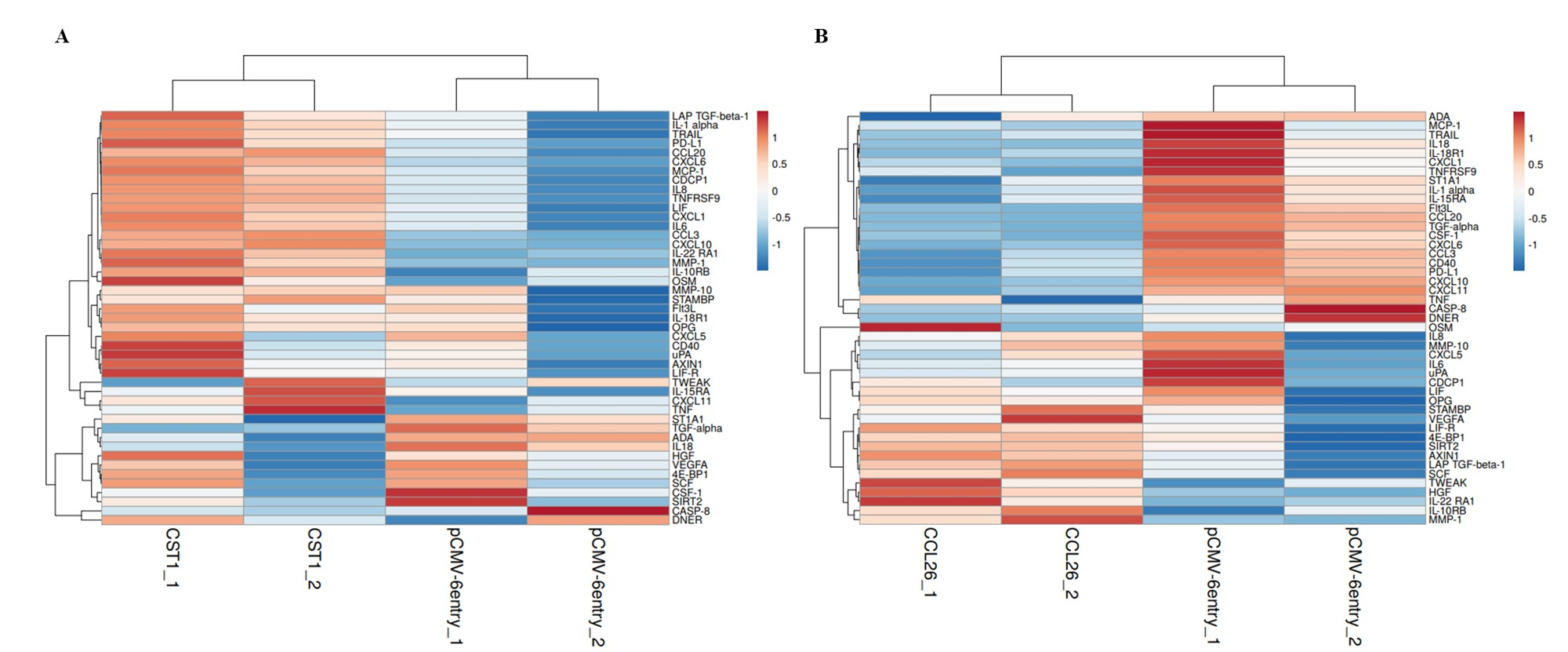


Supplementary Figure 3: Heatmap of the 45 differentially expressed proteins of A549 cells overexpressing (A) CST1 and (B) CCL26 versus the control vector pCMV-6entry.

| **Ensemble ID** | **Gene** | **Log_2_FC** | **FDR** |
| --- | --- | --- | --- |
| ENSG00000170373 | *CST1* | 8.25 | 0.000 |
| ENSG00000170369 | *CST2* | 3.23 | 0.000 |
| ENSG00000178685 | *PARP10* | -2.00 | 0.002 |
| ENSG00000134326 | *CMPK2* | -2.02 | 0.010 |
| ENSG00000135114 | *OASL* | -2.02 | 0.008 |
| ENSG00000185885 | *IFITM1* | -2.03 | 0.007 |
| ENSG00000100342 | *APOL1* | -2.06 | 0.004 |
| ENSG00000111335 | *OAS2* | -2.10 | 0.008 |
| ENSG00000259384 | *GH1* | -2.13 | 0.001 |
| ENSG00000078081 | *LAMP3* | -2.14 | 0.007 |
| ENSG00000165949 | *IFI27* | -2.16 | 0.005 |
| ENSG00000137198 | *GMPR* | -2.23 | 0.004 |
| ENSG00000224608 | *HLA-B* | -2.25 | 0.005 |
| ENSG00000141574 | *SECTM1* | -2.25 | 0.006 |
| ENSG00000130303 | *BST2* | -2.26 | 0.004 |
| ENSG00000010030 | *ETV7* | -2.29 | 0.007 |
| ENSG00000196684 | *HSH2D* | -2.30 | 0.006 |
| ENSG00000134321 | *RSAD2* | -2.32 | 0.006 |
| ENSG00000132530 | *XAF1* | -2.40 | 0.002 |
| ENSG00000131203 | *IDO1* | -2.41 | 0.004 |
| ENSG00000119922 | *IFIT2* | -2.47 | 0.005 |
| ENSG00000137403 | *HLA-F* | -2.47 | 0.001 |
| ENSG00000182393 | *IFNL1* | -2.63 | 0.003 |
| ENSG00000214872 | *SMTNL1* | -2.66 | 0.002 |
| ENSG00000183709 | *IFNL2* | -2.68 | 0.006 |
| ENSG00000171855 | *IFNB1* | -2.69 | 0.006 |
| ENSG00000182179 | *UBA7* | -3.06 | 0.000 |
| ENSG00000188282 | *RUFY4* | -4.11 | 0.007 |

Supplementary Table 1: Differentially expressed genes (DEG) after overexpression of CST1.

| **Ensemble ID** | **Gene** | **Log_2_FC** | **FDR** |
| --- | --- | --- | --- |
| ENSG00000006606 | *CCL26* | 7.90 | 0.000 |
| ENSG00000079385 | *CEACAM1* | -2.00 | 0.000 |
| ENSG00000123095 | *BHLHE41* | -2.01 | 0.003 |
| ENSG00000100918 | *REC8* | -2.03 | 0.000 |
| ENSG00000213689 | *TREX1* | -2.04 | 0.000 |
| ENSG00000125735 | *TNFSF14* | -2.04 | 0.000 |
| ENSG00000165731 | *RET* | -2.08 | 0.004 |
| ENSG00000129451 | *KLK10* | -2.10 | 0.009 |
| ENSG00000128335 | *APOL2* | -2.11 | 0.000 |
| ENSG00000237022 | *HLA-C* | -2.11 | 0.000 |
| ENSG00000002549 | *LAP3* | -2.12 | 0.000 |
| ENSG00000078018 | *MAP2* | -2.12 | 0.000 |
| ENSG00000223481 | *TAP2* | -2.13 | 0.000 |
| ENSG00000172183 | *ISG20* | -2.13 | 0.000 |
| ENSG00000140464 | *PML* | -2.18 | 0.000 |
| ENSG00000188313 | *PLSCR1* | -2.23 | 0.000 |
| ENSG00000129226 | *CD68* | -2.25 | 0.000 |
| ENSG00000120217 | *CD274* | -2.28 | 0.001 |
| ENSG00000108679 | *LGALS3BP* | -2.29 | 0.000 |
| ENSG00000106785 | *TRIM14* | -2.31 | 0.000 |
| ENSG00000144837 | *PLA1A* | -2.32 | 0.000 |
| ENSG00000163644 | *PPM1K* | -2.35 | 0.000 |
| ENSG00000115415 | *STAT1* | -2.35 | 0.000 |
| ENSG00000159403 | *C1R* | -2.35 | 0.000 |
| ENSG00000204540 | *PSORS1C1* | -2.36 | 0.001 |
| ENSG00000141497 | *ZMYND15* | -2.38 | 0.003 |
| ENSG00000185404 | *SP140L* | -2.39 | 0.000 |
| ENSG00000132109 | *TRIM21* | -2.43 | 0.000 |
| ENSG00000138642 | *HERC6* | -2.47 | 0.000 |
| ENSG00000198576 | *ARC* | -2.51 | 0.006 |
| ENSG00000117226 | *GBP3* | -2.53 | 0.000 |
| ENSG00000101347 | *SAMHD1* | -2.54 | 0.000 |
| ENSG00000122643 | *NT5C3A* | -2.55 | 0.000 |
| ENSG00000173193 | *PARP14* | -2.56 | 0.000 |
| ENSG00000206297 | *TAP1* | -2.57 | 0.000 |
| ENSG00000163840 | *DTX3L* | -2.63 | 0.000 |
| ENSG00000221963 | *APOL6* | -2.65 | 0.000 |
| ENSG00000265972 | *TXNIP* | -2.67 | 0.000 |
| ENSG00000025708 | *TYMP* | -2.71 | 0.000 |
| ENSG00000177989 | *ODF3B* | -2.78 | 0.000 |
| ENSG00000197646 | *PDCD1LG2* | -2.81 | 0.003 |
| ENSG00000181381 | *DDX60L* | -2.83 | 0.000 |
| ENSG00000130813 | *SHFL* | -2.83 | 0.000 |
| ENSG00000170581 | *STAT2* | -2.84 | 0.000 |
| ENSG00000100346 | *CACNA1I* | -2.84 | 0.003 |
| ENSG00000134339 | *SAA2* | -2.84 | 0.001 |
| ENSG00000203797 | *DDO* | -2.85 | 0.002 |
| ENSG00000244731 | *C4A* | -2.86 | 0.006 |
| ENSG00000106560 | *GIMAP2* | -2.86 | 0.003 |
| ENSG00000059378 | *PARP12* | -2.88 | 0.000 |
| ENSG00000185338 | *SOCS1* | -2.92 | 0.005 |
| ENSG00000132554 | *RGS22* | -2.92 | 0.000 |
| ENSG00000089127 | *OAS1* | -2.94 | 0.000 |
| ENSG00000135363 | *LMO2* | -2.98 | 0.000 |
| ENSG00000133321 | *PLAAT4* | -3.05 | 0.000 |
| ENSG00000155918 | *RAET1L* | -3.09 | 0.001 |
| ENSG00000172243 | *CLEC7A* | -3.12 | 0.006 |
| ENSG00000111331 | *OAS3* | -3.14 | 0.000 |
| ENSG00000136689 | *IL1RN* | -3.15 | 0.003 |
| ENSG00000156587 | *UBE2L6* | -3.17 | 0.000 |
| ENSG00000135899 | *SP110* | -3.21 | 0.000 |
| ENSG00000102524 | *TNFSF13B* | -3.22 | 0.001 |
| ENSG00000140853 | *NLRC5* | -3.25 | 0.000 |
| ENSG00000134716 | *CYP2J2* | -3.27 | 0.001 |
| ENSG00000138496 | *PARP9* | -3.28 | 0.000 |
| ENSG00000119698 | *PPP4R4* | -3.32 | 0.005 |
| ENSG00000205413 | *SAMD9* | -3.33 | 0.000 |
| ENSG00000152689 | *RASGRP3* | -3.34 | 0.000 |
| ENSG00000137198 | *GMPR* | -3.34 | 0.000 |
| ENSG00000142089 | *IFITM3* | -3.35 | 0.000 |
| ENSG00000242711 | *PSMB9* | -3.35 | 0.000 |
| ENSG00000137628 | *DDX60* | -3.35 | 0.000 |
| ENSG00000184979 | *USP18* | -3.39 | 0.000 |
| ENSG00000149131 | *SERPING1* | -3.39 | 0.001 |
| ENSG00000138646 | *HERC5* | -3.41 | 0.000 |
| ENSG00000068079 | *IFI35* | -3.44 | 0.000 |
| ENSG00000010030 | *ETV7* | -3.52 | 0.000 |
| ENSG00000163565 | *IFI16* | -3.52 | 0.000 |
| ENSG00000105559 | *PLEKHA4* | -3.53 | 0.000 |
| ENSG00000133106 | *EPSTI1* | -3.55 | 0.001 |
| ENSG00000241253 | *CFB* | -3.60 | 0.000 |
| ENSG00000141574 | *SECTM1* | -3.62 | 0.000 |
| ENSG00000164342 | *TLR3* | -3.64 | 0.000 |
| ENSG00000198574 | *SH2D1B* | -3.69 | 0.009 |
| ENSG00000130589 | *HELZ2* | -3.72 | 0.000 |
| ENSG00000230034 | *PSMB8* | -3.78 | 0.002 |
| ENSG00000108771 | *DHX58* | -3.82 | 0.000 |
| ENSG00000100342 | *APOL1* | -3.85 | 0.000 |
| ENSG00000132274 | *TRIM22* | -3.88 | 0.000 |
| ENSG00000271503 | *CCL5* | -3.90 | 0.000 |
| ENSG00000168016 | *TRANK1* | -3.99 | 0.000 |
| ENSG00000130775 | *THEMIS2* | -4.01 | 0.000 |
| ENSG00000177409 | *SAMD9L* | -4.09 | 0.000 |
| ENSG00000137752 | *CASP1* | -4.14 | 0.000 |
| ENSG00000107201 | *RIGI* | -4.15 | 0.000 |
| ENSG00000162654 | *GBP4* | -4.28 | 0.000 |
| ENSG00000178685 | *PARP10* | -4.35 | 0.000 |
| ENSG00000276561 | *IRF7* | -4.38 | 0.000 |
| ENSG00000115267 | *IFIH1* | -4.39 | 0.000 |
| ENSG00000121858 | *TNFSF10* | -4.41 | 0.000 |
| ENSG00000224608 | *HLA-B* | -4.45 | 0.000 |
| ENSG00000168961 | *LGALS9* | -4.51 | 0.000 |
| ENSG00000078081 | *LAMP3* | -4.52 | 0.000 |
| ENSG00000168062 | *BATF2* | -4.56 | 0.000 |
| ENSG00000183486 | *MX2* | -4.57 | 0.000 |
| ENSG00000169245 | *CXCL10* | -4.66 | 0.001 |
| ENSG00000229698 | *HLA-F* | -4.72 | 0.000 |
| ENSG00000182393 | *IFNL1* | -4.76 | 0.000 |
| ENSG00000128284 | *APOL3* | -4.79 | 0.000 |
| ENSG00000183709 | *IFNL2* | -4.82 | 0.000 |
| ENSG00000196684 | *HSH2D* | -4.84 | 0.000 |
| ENSG00000169248 | *CXCL11* | -4.85 | 0.002 |
| ENSG00000197110 | *IFNL3* | -4.86 | 0.001 |
| ENSG00000182179 | *UBA7* | -5.00 | 0.000 |
| ENSG00000157601 | *MX1* | -5.02 | 0.000 |
| ENSG00000126709 | *IFI6* | -5.06 | 0.000 |
| ENSG00000259384 | *GH1* | -5.06 | 0.000 |
| ENSG00000117228 | *GBP1* | -5.16 | 0.000 |
| ENSG00000185885 | *IFITM1* | -5.26 | 0.000 |
| ENSG00000137965 | *IFI44* | -5.27 | 0.000 |
| ENSG00000132530 | *XAF1* | -5.33 | 0.000 |
| ENSG00000187608 | *ISG15* | -5.36 | 0.000 |
| ENSG00000135114 | *OASL* | -5.37 | 0.000 |
| ENSG00000214872 | *SMTNL1* | -5.41 | 0.000 |
| ENSG00000130303 | *BST2* | -5.51 | 0.000 |
| ENSG00000119917 | *IFIT3* | -5.56 | 0.000 |
| ENSG00000185745 | *IFIT1* | -5.58 | 0.000 |
| ENSG00000134326 | *CMPK2* | -5.66 | 0.000 |
| ENSG00000111335 | *OAS2* | -5.75 | 0.000 |
| ENSG00000131203 | *IDO1* | -5.82 | 0.000 |
| ENSG00000171855 | *IFNB1* | -5.83 | 0.000 |
| ENSG00000119922 | *IFIT2* | -6.14 | 0.000 |
| ENSG00000188282 | *RUFY4* | -6.28 | 0.001 |
| ENSG00000137959 | *IFI44L* | -6.31 | 0.000 |
| ENSG00000134321 | *RSAD2* | -6.96 | 0.000 |
| ENSG00000110446 | *SLC15A3* | -7.09 | 0.000 |
| ENSG00000136514 | *RTP4* | -7.16 | 0.000 |
| ENSG00000165949 | *IFI27* | -7.18 | 0.000 |

Supplementary Table 2: Differentially expressed genes (DEG) after overexpression of CCL26.

| Proteins included in the analysis (= 45) | Proteins not included in the analysis  (= 47) |
| --- | --- |
| IL8 (CXCL8) | CD8A |
| VEGFA | MCP-3 (CCL7) |
| CDCP1 | GDNF |
| OPG | CD244 |
| LAP TGF-beta-1 | IL7 |
| uPA (PLAU) | IL-17C |
| IL6 | IL-20RA |
| MCP-1 (CCL2) | CXCL9 |
| CXCL11 | CST5 |
| AXIN1 | IL-2RB |
| TRAIL (TNFSF10) | IL2 |
| IL-1 alpha | TSLP |
| OSM | CCL4 |
| CXCL1 | CD6 |
| SCF | SLAMF1 |
| IL18 | MCP-4 (CCL13) |
| TGF-alpha | CCL11 |
| MMP-1 | TNFSF14 |
| LIF-R | FGF-23 |
| IL-15RA | IL-10RA |
| IL-10RB | FGF-5 |
| IL-22 RA1 | FGF-21 |
| IL-18R1 | CCL19 |
| PD-L1 | Beta-NGF |
| CXCL5 | TRANCE (TNFSF11) |
| HGF | IL-12B |
| MMP-10 | IL-24 |
| TNF | IL13 |
| CCL3 | ARTN |
| Flt3L | IL10 |
| CXCL6 | CCL23 |
| CXCL10 | CD5 |
| 4E-BP1 | IL-20 |
| SIRT2 | CCL28 |
| DNER | EN-RAGE (S100A12) |
| CD40 | IL33 |
| LIF | IFN-gamma |
| CASP-8 | FGF-19 |
| TNFRSF9 | IL4 |
| TWEAK (TNFSF12) | NRTN |
| CCL20 | MCP-2 (CCL8) |
| ST1A1 | CCL25 |
| STAMBP | CX3CL1 |
| ADA | TNFRSF9 |
| CSF-1 | NT-3 |
|  | IL5 |
|  | TNFB (LTA) |

Supplementary Table 3: Included and excluded proteins from the OLINK analysis. Proteins with a sample missing data frequency over 15% (below the Limit of Detection (LOD)) were excluded from the analysis.

| **Assay** | **Mean overexpressing *CST1*** | **Mean empty control vector** | **log_2_FC** | **p-value** |
| --- | --- | --- | --- | --- |
| 4E-BP1 | 9.07 | 9.11 | -0.04 | 0.765 |
| ADA | 9.41 | 9.81 | -0.40 | 0.081 |
| AXIN1 | 8.40 | 8.22 | 0.18 | 0.363 |
| CASP-8 | 5.72 | 6.56 | -0.84 | 0.352 |
| CCL20 | 9.69 | 9.16 | 0.54 | 0.058 |
| CCL3 | 7.57 | 5.58 | 1.99 | 0.004 |
| CD40 | 12.25 | 12.16 | 0.09 | 0.565 |
| CDCP1 | 9.44 | 8.91 | 0.52 | 0.069 |
| CSF-1 | 8.20 | 8.33 | -0.13 | 0.335 |
| CXCL1 | 11.51 | 11.03 | 0.48 | 0.091 |
| CXCL10 | 6.99 | 6.25 | 0.74 | 0.004 |
| CXCL11 | 9.31 | 8.92 | 0.39 | 0.136 |
| CXCL5 | 12.55 | 12.53 | 0.02 | 0.834 |
| CXCL6 | 4.22 | 3.88 | 0.34 | 0.021 |
| DNER | 0.47 | 0.43 | 0.04 | 0.783 |
| Flt3L | 5.39 | 5.33 | 0.07 | 0.548 |
| HGF | 0.73 | 0.77 | -0.04 | 0.898 |
| IL-1 alpha | 2.80 | 2.31 | 0.50 | 0.135 |
| IL-10RB | 2.00 | 1.68 | 0.32 | 0.071 |
| IL-15RA | 1.80 | 1.66 | 0.15 | 0.362 |
| IL18 | 7.62 | 8.12 | -0.50 | 0.055 |
| IL-18R1 | 6.54 | 6.28 | 0.25 | 0.303 |
| IL-22 RA1 | 4.83 | 4.42 | 0.41 | 0.019 |
| IL6 | 8.94 | 8.47 | 0.47 | 0.089 |
| IL8 | 10.97 | 10.44 | 0.53 | 0.055 |
| LAP TGF-beta-1 | 4.83 | 4.43 | 0.41 | 0.174 |
| LIF | 4.15 | 3.87 | 0.29 | 0.107 |
| LIF-R | 1.07 | 0.83 | 0.24 | 0.241 |
| MCP-1 | 10.93 | 10.39 | 0.54 | 0.065 |
| MMP-1 | 3.79 | 3.38 | 0.41 | 0.033 |
| MMP-10 | 0.49 | 0.35 | 0.14 | 0.463 |
| OPG | 1.78 | 1.62 | 0.16 | 0.392 |
| OSM | -0.67 | -0.85 | 0.18 | 0.142 |
| PD-L1 | 8.28 | 7.90 | 0.38 | 0.075 |
| SCF | 3.96 | 3.97 | -0.01 | 0.911 |
| SIRT2 | 4.46 | 4.54 | -0.08 | 0.709 |
| ST1A1 | 4.87 | 5.15 | -0.28 | 0.280 |
| STAMBP | 9.03 | 8.85 | 0.18 | 0.299 |
| TGF-alpha | 2.85 | 3.35 | -0.51 | 0.025 |
| TNF | 0.16 | 0.01 | 0.15 | 0.260 |
| TNFRSF9 | 8.03 | 7.56 | 0.47 | 0.049 |
| TRAIL | 8.18 | 7.89 | 0.30 | 0.186 |
| TWEAK | 3.39 | 3.38 | 0.01 | 0.948 |
| uPA | 11.81 | 11.70 | 0.11 | 0.471 |
| VEGFA | 9.09 | 9.12 | -0.04 | 0.589 |

Supplementary Table 4: Protein expression results of the OLINK analysis. The Target 96 inflammation panel from OLINK measured the cell lysate of A549 cells overexpressing CST1. The protein expression was compared to the cells that were transfected with the empty control vector. All samples were measured in duplicates. The protein expression values are presented as log_2_FC and p-values were calculated with t-test.

| **Assay** | **Mean overexpressing *CCL26*** | **Mean empty control vector** | **log_2_FC** | **p-value** |
| --- | --- | --- | --- | --- |
| 4E-BP1 | 9.19 | 9.11 | 0.08 | 0.310 |
| ADA | 9.69 | 9.81 | -0.12 | 0.277 |
| AXIN1 | 8.58 | 8.22 | 0.36 | 0.107 |
| CASP-8 | 5.59 | 6.56 | -0.97 | 0.301 |
| CCL20 | 7.52 | 9.16 | -1.64 | 0.006 |
| CCL3 | 4.46 | 5.58 | -1.12 | 0.031 |
| CD40 | 11.50 | 12.16 | -0.66 | 0.045 |
| CDCP1 | 8.86 | 8.91 | -0.05 | 0.770 |
| CSF-1 | 7.90 | 8.33 | -0.43 | 0.043 |
| CXCL1 | 10.75 | 11.03 | -0.28 | 0.178 |
| CXCL10 | 5.17 | 6.25 | -1.07 | 0.004 |
| CXCL11 | 7.20 | 8.92 | -1.73 | 0.013 |
| CXCL5 | 12.52 | 12.53 | -0.01 | 0.879 |
| CXCL6 | 3.63 | 3.88 | -0.25 | 0.032 |
| DNER | 0.16 | 0.43 | -0.27 | 0.135 |
| Flt3L | 4.75 | 5.33 | -0.58 | 0.020 |
| HGF | 1.88 | 0.77 | 1.10 | 0.038 |
| IL-1 alpha | 1.67 | 2.31 | -0.64 | 0.103 |
| IL-10RB | 1.91 | 1.68 | 0.23 | 0.153 |
| IL-15RA | 1.37 | 1.66 | -0.29 | 0.141 |
| IL18 | 7.87 | 8.12 | -0.25 | 0.089 |
| IL-18R1 | 5.87 | 6.28 | -0.41 | 0.148 |
| IL-22 RA1 | 4.79 | 4.42 | 0.37 | 0.105 |
| IL6 | 8.43 | 8.47 | -0.04 | 0.796 |
| IL8 | 10.48 | 10.44 | 0.04 | 0.778 |
| LAP TGF-beta-1 | 4.83 | 4.43 | 0.41 | 0.127 |
| LIF | 3.90 | 3.87 | 0.03 | 0.766 |
| LIF-R | 0.99 | 0.83 | 0.15 | 0.230 |
| MCP-1 | 10.24 | 10.39 | -0.15 | 0.341 |
| MMP-1 | 3.62 | 3.38 | 0.24 | 0.058 |
| MMP-10 | 0.40 | 0.35 | 0.05 | 0.789 |
| OPG | 1.71 | 1.62 | 0.09 | 0.600 |
| OSM | -0.76 | -0.85 | 0.09 | 0.669 |
| PD-L1 | 7.24 | 7.90 | -0.66 | 0.039 |
| SCF | 4.15 | 3.97 | 0.18 | 0.128 |
| SIRT2 | 4.85 | 4.54 | 0.31 | 0.228 |
| ST1A1 | 4.94 | 5.15 | -0.21 | 0.144 |
| STAMBP | 9.04 | 8.85 | 0.19 | 0.328 |
| TGF-alpha | 2.48 | 3.35 | -0.87 | 0.009 |
| TNF | -0.13 | 0.01 | -0.14 | 0.396 |
| TNFRSF9 | 7.34 | 7.56 | -0.22 | 0.204 |
| TRAIL | 7.68 | 7.89 | -0.21 | 0.276 |
| TWEAK | 3.56 | 3.38 | 0.18 | 0.192 |
| uPA | 11.67 | 11.70 | -0.03 | 0.723 |
| VEGFA | 9.20 | 9.12 | 0.07 | 0.297 |

Supplementary Table 5: Protein expression results of the OLINK analysis. The Target 96 inflammation panel from OLINK measured the cell lysate of A549 cells overexpressing CCL26. The protein expression was compared to the cells that were transfected with the empty control vector. All samples were measured in duplicates. The protein expression values are presented as log_2_FC and p-values were calculated with t-test.
